# Open and Percutaneous Fixation of Traumatic Sacral Fracture–Dislocation with Spinopelvic Dissociation: Two Adolescent Cases and a Systematic Literature Review

**DOI:** 10.3390/jcm15134914

**Published:** 2026-06-24

**Authors:** Angelo Carosini, Calogero Velluto, Maria Ilaria Borruto, Laura Scaramuzzo, Maurizio Genitiempo, Felice Minutillo, Giulio Maccauro, Luca Proietti

**Affiliations:** Department of Aging, Orthopaedic and Rheumatological Sciences, Fondazione Policlinico Universitario Agostino Gemelli IRCCS, Largo A. Gemelli, 8, 00168 Rome, Italy; angelo.carosini@gmail.com (A.C.); maria.ilaria.borruto@gmail.com (M.I.B.); laura.scaramuzzo@policlinicogemelli.it (L.S.); maurizio.genitiempo@policlinicogemelli.it (M.G.); felice.minutillo@policlinicogemelli.it (F.M.); giulio.maccauro@policlinicogemelli.it (G.M.); luca.proietti@policlinicogemelli.it (L.P.)

**Keywords:** spinopelvic dissociation, sacral fracture–dislocation, lumbopelvic fixation, open reduction and internal fixation, percutaneous fixation, adolescent trauma

## Abstract

**Background:** Spinopelvic dissociation secondary to sacral fracture–dislocation is a rare but severe injury, most often resulting from high-energy trauma. Management remains challenging, particularly in adolescents, and the optimal choice between open and percutaneous fixation is still debated. **Methods:** We present two adolescent cases of traumatic sacral fracture–dislocation with spinopelvic dissociation, one treated with percutaneous fixation and one with open lumbopelvic stabilization both with the use of navigation. The systematic literature review included 29 published studies. Together with the present two-patient case series, the overall analysis comprised 30 studies/series and 739 patients. Data on demographics, mechanisms of injury, neurological involvement, treatment strategies, and outcomes were extracted and analyzed. **Results:** Case 1 (18 years) was managed with closed reduction and percutaneous fixation, achieving complete neurological and functional recovery at 6 months. Case 2 (14 years) underwent open reduction, decompression, and lumbopelvic fixation, with favorable radiological outcomes but residual sphincter dysfunction at follow-up. In the literature, the weighted mean age was 40.6 years (range 5–91), with 48.6% presenting neurological deficits, most frequently cauda equina syndrome. Surgical management was performed in nearly all cases, with mean time to fixation ranging from 3.6 to 8.6 days. Open techniques were predominantly used in patients with severe displacement or neurological compromise, whereas percutaneous fixation was associated with reduced surgical morbidity and satisfactory neurological recovery in selected patients. Permanent bladder and bowel dysfunction persisted in up to 33% of cases. **Conclusions:** Spinopelvic dissociation following sacral fracture–dislocation remains a rare and highly unstable injury with frequent neurological impairment. Early surgical stabilization may be beneficial when the patient’s clinical condition permits, and the choice between open and percutaneous fixation should be individualized according to fracture morphology, neurological status, and the need for direct decompression. Our adolescent cases highlight both the potential for complete recovery and the risk of residual dysfunction, reflecting the complexity of these injuries.

## 1. Introduction

Sacral fractures associated with spinopelvic dissociation are uncommon, representing approximately 2–3% of sacral fractures, but they are clinically important because they can produce marked lumbopelvic instability [1]. These injuries are frequently associated with vascular, visceral, neurological, and ambulatory impairment [2] and typically result from high-energy trauma, particularly falls from height and road-traffic accidents [3]. Concomitant pelvic, spinal, extremity, thoracic, abdominal, and cranial injuries are common and may complicate early diagnosis and management [2]. Neurological involvement ranges from isolated radiculopathy to cauda equina syndrome, and recovery remains variable [4]. Greater kyphotic deformity, particularly angulation of 20° or more, has been associated with an increased risk of neurological injury [5].

Diagnosis and definitive treatment may be delayed in polytraumatized patients because life-threatening injuries often take priority and fracture morphology may be difficult to recognize [3]. Although intervention within the early post-traumatic period has been associated with favorable neurological and functional outcomes, the optimal timing remains uncertain and must be adapted to the patient’s overall condition [3]. Delayed stabilization may contribute to malunion, persistent neural compression, chronic pain, prolonged hospitalization, and functional impairment [4].

The choice of surgical strategy remains individualized. Open reduction and fixation may be appropriate when substantial displacement, canal compromise, or direct neural decompression is present, whereas percutaneous fixation may reduce surgical morbidity in selected injuries that are amenable to indirect reduction. Evidence in adolescents is particularly limited, and differences in initial injury severity make direct comparison between techniques difficult. This study therefore describes two adolescent cases managed using different surgical strategies and systematically reviews the available evidence on injury characteristics, surgical decision-making, complications, and neurological and functional outcomes. It was not designed to determine the superiority of open or percutaneous fixation.

## 2. Material and Methods

### 2.1. Search Strategy

**Figure 1:**
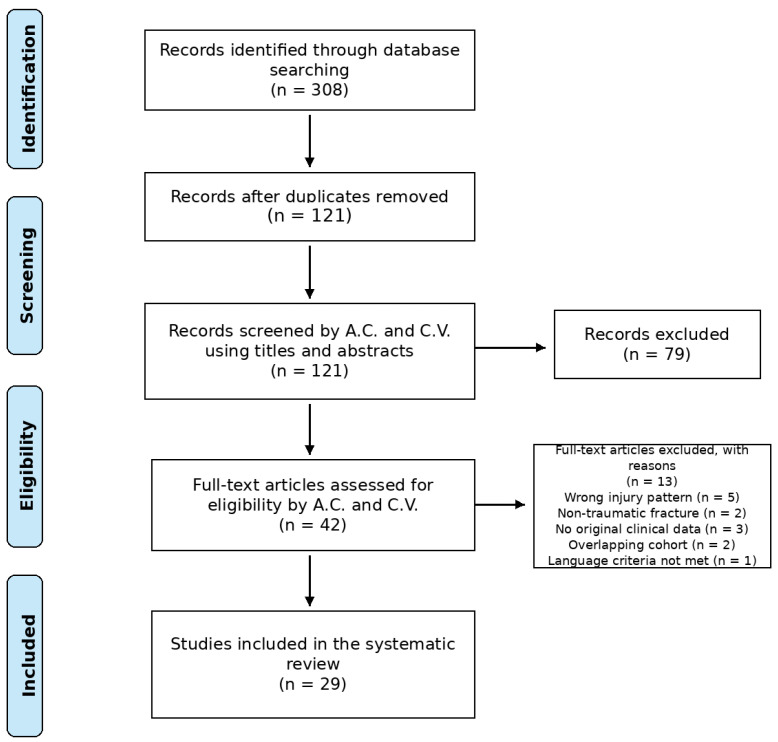
PRISMA flowchart.

A systematic literature search was performed in accordance with the PRISMA (Preferred Reporting Items for Systematic Reviews and Meta-Analyses) guidelines [6,7]. The completed PRISMA 2020 checklist is provided as Supplementary Table S1. The electronic databases PubMed/MEDLINE, Scopus, and Web of Science were searched from January 2000 to August 2025 in order to identify all relevant studies reporting cases of traumatic sacral fracture–dislocation associated with spinopelvic or lumbopelvic dissociation. The following keywords and free-text terms were used in various combinations with Boolean operators (“AND”, “OR”): “sacral fracture-dislocation”, “spinopelvic dissociation”, “lumbopelvic dissociation”, “traumatic lumbosacral instability”, and “U-shaped sacral fracture”. No restrictions were applied regarding patient age, sex, or study type to ensure inclusion of both adult and pediatric/adolescent cases. The search was restricted to articles published in English or with an English abstract available. Additional relevant studies were identified through manual screening of the reference lists of included articles. A flowchart is reported in Figure 1.

The protocol of this systematic review was not prospectively registered, and no formal review protocol was prepared or published.

The complete database-specific search strategies were as follows:

PubMed/MEDLINE: (“sacral fracture-dislocation” [Title/Abstract] OR “spinopelvic dissociation” [Title/Abstract] OR “lumbopelvic dissociation” [Title/Abstract] OR “traumatic lumbosacral instability” [Title/Abstract] OR “U-shaped sacral fracture” [Title/Abstract]) AND (trauma*[Title/Abstract] OR traumatic [Title/Abstract]).

Scopus: TITLE-ABS-KEY (“sacral fracture-dislocation” OR “spinopelvic dissociation” OR “lumbopelvic dissociation” OR “traumatic lumbosacral instability” OR “U-shaped sacral fracture”) AND TITLE-ABS-KEY (trauma* OR traumatic).

Web of Science: TS = (“sacral fracture-dislocation” OR “spinopelvic dissociation” OR “lumbopelvic dissociation” OR “traumatic lumbosacral instability” OR “U-shaped sacral fracture”) AND TS = (trauma* OR traumatic).

Searches were limited to publications from January 2000 to August 2025 and to articles published in English or with an English abstract available. The final search was performed on 30 August 2025.

### 2.2. Inclusion and Exclusion Criteria

All studies reporting traumatic sacral fracture–dislocations with spinopelvic or lumbopelvic dissociation were considered eligible, regardless of patient age, sex, or sample size. We included case reports, case series, retrospective and prospective cohort studies, and clinical trials that provided sufficient demographic, clinical, radiological, therapeutic, or outcome data. Exclusion criteria were non-traumatic sacral fractures (e.g., pathological, insufficiency, or stress fractures); cadaveric or purely biomechanical studies without clinical data; reviews, editorials, expert opinions, and conference abstracts lacking original data; and articles not available in English or without an English abstract. When multiple publications from the same cohort were identified, the most complete or recent report was included.

### 2.3. Data Collection

Two reviewers independently screened all titles and abstracts obtained through the initial search. Full texts of potentially relevant articles were then assessed in detail to determine eligibility. Any disagreements regarding study selection were resolved by consensus and, when necessary, by consultation with a senior reviewer. The information recorded encompassed general study characteristics (such as first author, year of publication, country of origin, study design, and level of evidence), as well as details on the patient population, including sample size, demographic features, and age distribution. Particular attention was given to the description of the injury, with documentation of fracture classification systems applied, presence of associated injuries, and the incidence and type of neurological deficits. Management strategies were carefully reviewed, distinguishing between conservative and surgical approaches, with further specification of the surgical techniques adopted and the timing of intervention. Outcome measures, including functional recovery, neurological improvement, complication rates, and length of follow-up, were also collected when reported. Quantitative data, such as means, ranges, and standard deviations, were extracted when available. In cases where data presentation was incomplete or ambiguous, information was obtained through careful analysis of the text, figures, and Supplementary Materials. All retrieved references were imported into Zotero (Zotero version 9.0.4, Corporation for Digital Scholarship, Vienna, VA, USA). Duplicate records were initially identified using Zotero and were subsequently verified and removed manually by the authors through comparison of titles, authors, publication years, abstracts, and, when necessary, full manuscripts. Of the 308 records initially identified, 187 duplicate records were removed, leaving 121 records for title and abstract screening.

At the full-text eligibility stage, 13 articles were excluded for the following reasons: wrong injury pattern (*n* = 5), non-traumatic fracture (*n* = 2), no original clinical data (*n* = 3), overlapping cohort (*n* = 2), and language criteria not met (*n* = 1).

### 2.4. Statistical Analysis

Given the heterogeneity of the available studies in terms of design, patient populations, and outcome measures, a formal meta-analysis was not feasible. Therefore, data were synthesized using a descriptive approach. Continuous variables, when reported, were summarized as means with corresponding ranges or standard deviations, whereas categorical variables were presented as absolute numbers and percentages. The results from individual studies were then qualitatively compared and integrated in order to highlight recurring patterns regarding epidemiology, management strategies, and clinical outcomes.

### 2.5. Risk of Bias and Methodological Quality Assessment

Two reviewers independently assessed the methodological quality of the included studies using design-specific Joanna Briggs Institute critical appraisal tools. Case reports were evaluated using the JBI Critical Appraisal Checklist for Case Reports, case series using the JBI Critical Appraisal Checklist for Case Series, and observational comparative or cohort studies using the JBI Critical Appraisal Checklist for Cohort Studies. Each domain was judged as low concern, moderate concern, high concern, unclear, or not applicable, according to the completeness and methodological safeguards reported in the source article. Particular attention was given to participant selection, diagnostic and outcome ascertainment, completeness and duration of follow-up, control of confounding, and completeness of reporting. Disagreements were resolved by discussion and consensus. No study was excluded solely on the basis of methodological quality; the appraisal was used to inform interpretation of the evidence. Study-level judgments are reported in Supplementary Table S2.

## 3. Case Series

### 3.1. Case 1

An 18-year-old female was admitted to our Emergency Department following a motor-vehicle accident, in which she was a passenger on a motorcycle. On arrival, she was alert and oriented, reporting a cranial trauma but unable to recall the dynamics of the event. Neurological examination revealed hypoesthesia in the lateral region of the left ankle and foot, without motor or sphincter deficits. Past medical history was unremarkable.

**Figure 2:**
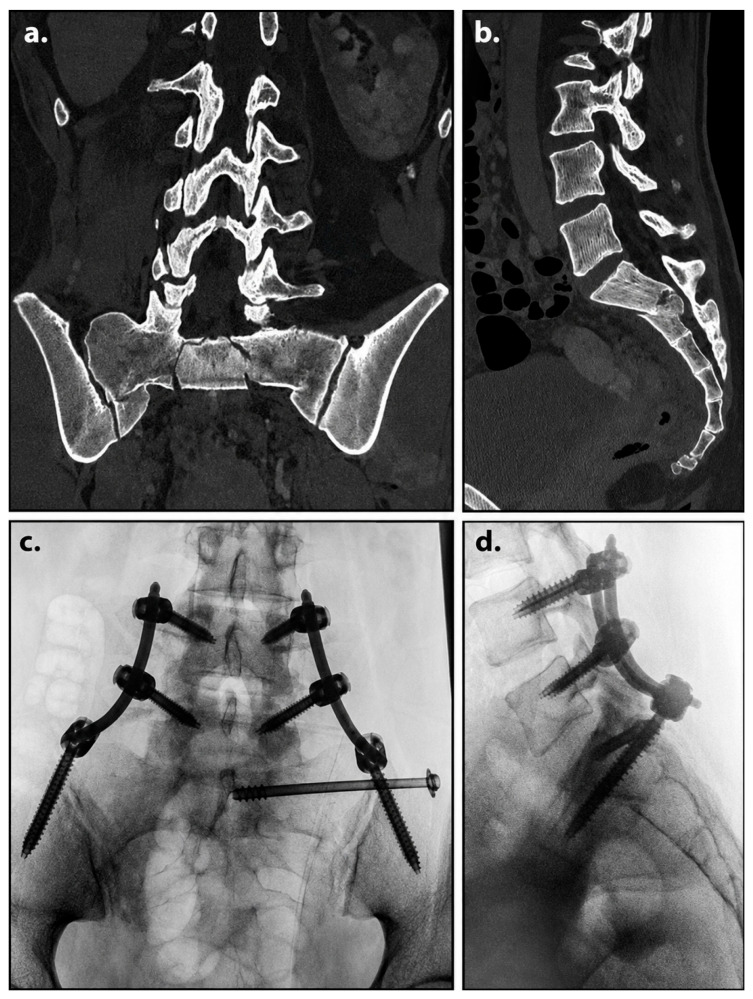
Case 1: Preoperative and postoperative imaging of Case 1. (**a**) Coronal CT reconstruction showing a U-shaped sacral fracture with lumbopelvic dissociation. (**b**) Sagittal CT reconstruction demonstrating kyphotic angulation of approximately 45° at S2 with involvement of the L5–S1 facet joints. (**c**) Postoperative anteroposterior radiograph after closed reduction and percutaneous fixation, showing bilateral pedicle screws at L5 and iliac screws connected by rods with a trans-sacral screw. (**d**) Postoperative lateral radiograph confirming restoration of alignment and stable construct positioning.

Computed tomography (CT) demonstrated a U-shaped fracture of the sacral body, with a transverse fracture line at the S2 level, as shown in Figure 2. The fracture was impacted and displaced, with approximately 45° of kyphotic angulation and involvement of the L5–S1 facet joints. Based on these findings, the patient was diagnosed with spinopelvic dissociation and admitted to our Spine Surgery Unit for stabilization and preoperative planning. After approximately 48 h, she underwent closed reduction and percutaneous osteosynthesis. The procedure was performed under continuous neuromonitoring with the aid of the Pulse^®^ NuVasive navigation system to support accurate screw placement and limit intraoperative radiation exposure. Reduction was achieved indirectly, and fixation was performed using a triangular lumbopelvic construct. Estimated blood loss was minimal (<100 mL), and no intraoperative complications occurred.

The postoperative course was uneventful. The patient was mobilized early, and full weight-bearing was allowed during hospitalization. She was discharged after 7 days in good general condition. At the 3-month follow-up, clinical evaluation revealed complete resolution of the hypoesthesia previously noted in the lateral aspect of the left ankle and foot. Radiographic assessment showed maintenance of reduction, with no evidence of implant loosening or loss of alignment. The patient reported only mild low back pain (VAS 2/10) after prolonged walking, which did not interfere with daily activities. Functional evaluation according to the Oswestry Disability Index (ODI) demonstrated minimal disability (ODI 8%).

At the 6-month follow-up, the patient had returned to her usual activities, including light sports. Neurological examination was normal, with no motor or sensory deficits. CT confirmed solid fracture healing, preserved alignment, and intact instrumentation. The patient reported no residual pain (VAS 0/10) and minimal disability (ODI 2%). No complications, including infection, hardware failure, or secondary deformity, were observed. This case illustrates the feasibility of early minimally invasive stabilization in a selected adolescent patient with spinopelvic dissociation. Navigation-assisted percutaneous fixation supported accurate implant placement and early mobilization and was associated with favorable neurological and functional recovery.

### 3.2. Case 2

**Figure 3:**
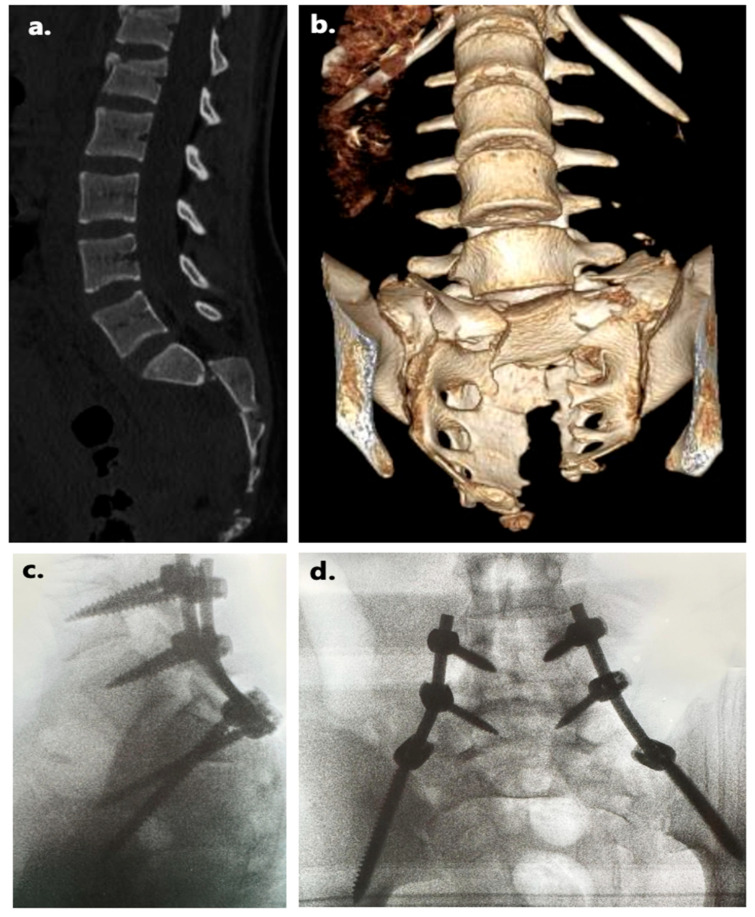
Case 2: (**a**) Sagittal CT scan showing a comminuted burst-type sacral fracture extending from the sacral base to the lower body, with spondyloptosis of S1 over S2. (**b**) Three-dimensional CT reconstruction illustrating the markedly displaced vertical fracture line on the left side and the associated pelvic deformity. (**c**) Postoperative lateral radiograph after open reduction and lumbopelvic fixation, demonstrating bilateral pedicle screws in L4–L5 and iliac screws without transverse connector due to sacral comminution. (**d**) Postoperative anteroposterior radiograph confirming maintenance of reduction and stable implant positioning.

A 14-year-old female was admitted to our Level I Emergency Department following a fall from height in the context of a suicide attempt. On arrival, she was conscious and hemodynamically stable, although she exhibited signs of polytrauma. Neurological examination revealed hypoesthesia in the lateral aspect of the left ankle and foot, without motor weakness. No sphincter dysfunction was reported at the presentation. Her past medical history was unremarkable. Computed tomography (CT) demonstrated a comminuted burst-type fracture of the sacrum extending from the sacral base to the lower sacral body, involving all sacral foramina, as shown in Figure 3. On the left side, the main vertical fracture line was markedly displaced and diastatic. Associated findings included bilateral fractures of the L5 transverse processes and spondyloptosis of S1 over S2. A concomitant fracture of the proximal third of the left femur was also identified. After initial stabilization in the Emergency Department, the patient was transferred to the pediatric intensive care unit (PICU). Approximately 48 h after admission, the patient underwent open reduction, decompression, and lumbopelvic fixation. The procedure was performed under continuous neuromonitoring and with the aid of the Pulse^®^ navigation system (NuVasive, Inc., San Diego, CA, USA), which was used to support accurate screw placement and limit intraoperative radiation exposure. Fixation was achieved with pedicle screws at L4 and L5 bilaterally and bilateral iliac screws. No transverse connector was used due to the high degree of sacral comminution, which precluded anatomical reduction in the smaller fragments. Intraoperatively, a lesion of a distal sacral root was identified and treated with TachoSil^®^ and VISTASEAL™ (Ethicon, Raritan, NJ, USA). Estimated blood loss was less than 300 mL, and no intraoperative complications occurred. The postoperative course was uneventful. Early mobilization was delayed due to the associated femoral fracture, which was surgically treated four days later. The patient was discharged from the PICU after 7 days and subsequently from our ward in a stable condition. At 3-month follow-up, clinical evaluation showed resolution of the left-sided hypoesthesia. Radiographs demonstrated maintenance of reduction and intact instrumentation, without loss of alignment. The patient reported mild low back pain only after prolonged walking (VAS 2/10), with minimal disability according to the Oswestry Disability Index (ODI 8%). At 6 months, she had resumed normal daily activities, including light sports. Neurological examination was normal for motor and sensory function; however, the patient reported mild persistent urinary urgency, consistent with residual sphincter dysfunction. CT confirmed solid fracture healing with preserved alignment and no evidence of implant loosening or infection. The final functional outcome was excellent (ODI 4%), though not indicative of complete recovery due to the residual bladder symptomatology. This case illustrates the potential role of early surgical stabilization and navigation-assisted techniques in complex adolescent spinopelvic dissociation. Despite favorable radiological and functional outcomes, residual neurological impairment may persist, particularly in the presence of severe sacral comminution and intraoperative root injury.

## 4. Literature Review

### 4.1. Methodological Quality of the Included Studies

Methodological quality was heterogeneous. Of the 29 included studies, 1 was judged to have low overall methodological concern, 24 moderate concern, and 4 high concern. Most evidence derived from retrospective case series and case reports. The most frequent limitations were unclear consecutive or complete inclusion, small sample size, heterogeneous definitions of neurological and functional outcomes, incomplete standardized follow-up, and limited reporting of adverse events. Comparative studies were particularly affected by confounding by indication, because open reduction and decompression were generally selected for more severe injuries, whereas percutaneous fixation was more commonly used in less displaced injuries without a clear need for direct decompression. Accordingly, unadjusted differences between surgical groups should not be interpreted as evidence of superiority. Study-level judgments are presented in Supplementary Table S2.

### 4.2. Epidemiology and Mechanism of Injury

The systematic literature review included 29 published studies as reported in Table 1. These studies were analyzed alongside the present two-patient case series, resulting in a total of 30 studies/series and 739 patients with spinopelvic dissociation [1,8,9,10,11,12,13,14,15,16,17,18,19,20,21,22,23,24,25,26,27,28,29,30,31,32,33,34,35]. The weighted mean age across the studies that reported a mean was 40.6 years, with reported ages spanning from 5 to 91 years [1,9,10,11,12,13,14,15,16,17,18,19,20,21,22,23,24,25,26,27,28,29,30,31,32,33,34,35,36]. Sex was explicitly stated in a subset of cohorts totaling 427 patients across 19 studies, with 244 males and 183 females [1,9,10,11,12,13,14,15,16,17,18,19,20,21,22,23,24,25,26,27,28,29,30,31,32,33,34,35,36]. Mechanism of injury was detailed in cohorts encompassing 262 patients. Within this subset, falls from height accounted for 114 cases (43.5%), road-traffic accidents for 103 cases (39.3%), crush or compression injuries for 16 cases (6.1%), and other mechanisms for 29 cases (11.1%). Overall, the injury pattern was consistently related to high-energy trauma. Several authors provided precise distributions. Young et al. [11], in a cohort of 40 patients with a mean age of 40 years (range 16–73), reported 20 motor-vehicle accidents, 9 falls from height, 2 crush injuries, and 9 cases due to other mechanisms, with all patients treated using open lumbopelvic fixation. In a separate cohort of 53 patients, Young et al. [15] described percutaneous posterior pelvic fixation as the main approach, with 15 patients presenting neurological deficits, typically radicular pain or paresthesias. Taylor et al. [8] analyzed 48 patients divided into two groups according to surgical technique, with 21 treated with open fixation and 27 with percutaneous fixation, reporting mean ages of 49.2 and 42.6 years, respectively; notably, seven cases presented with cauda equina syndrome, of which three were treated with open decompression and four with a percutaneous approach. Elsherif et al. [16] described 33 patients, with 21 sustaining falls from height and 12 involved in motor-vehicle accidents. Tian et al. [21] analyzed 28 patients with a mean age of 30 years (range 16–59), identifying 19 motor-vehicle accidents and 9 falls from height as causative mechanisms. Jindal et al. [25] reported 22 patients, 18 due to road-traffic accidents and 4 to falls from height, while Miyamoto et al. [26] reported an equal distribution of 3 motor-vehicle accidents and 3 falls from height in their series of 6 patients. Park et al. [33], in a large series of 71 patients, recorded 35 traffic accidents, 13 fall-related accidents, 12 slips, 5 patients buried under heavy loads, and an additional 3 falls from height. Shi et al. [17], in one of the largest cohorts with 138 patients, reported 12 falls, 9 motor-vehicle accidents, and 9 compression injuries, with the remaining cases not specified in detail; the mean age was 40.7 years (range 16–68).

### 4.3. Clinical Presentation and Diagnosis

Neurological involvement emerged as a consistent hallmark of spinopelvic dissociation across the studies included in this review. Out of the 739 patients analyzed, neurological data were available for 430, of whom 209 (48.6%) presented with neurological deficits ranging from incomplete radiculopathies to full-blown cauda equina syndrome. Taylor et al. [8], in a cohort of 48 patients with a mean age of 49.2 years in the open group and 42.6 years in the percutaneous group, reported seven cases of cauda equina syndrome, three of which were treated with open decompression and four with percutaneous fixation. Young et al. [11], analyzing 40 patients with a mean age of 40 years (16–73), described 3 patients with isolated sensory deficits, 5 with motor deficits, and 12 with combined neurological impairment, underlining the heterogeneity of presentation. In another series, Young et al. reported 15 neurological deficits out of 53 patients, mostly characterized by radicular pain and paresthesias [15]. Shi et al., in the largest cohort of 138 patients with a mean age of 40.7 years (16–68), reported nine cases of nerve injury, without specifying the exact distribution between motor and sensory involvement [17]. Tian et al., in a series of 28 patients with a mean age of 30 years (16–59), recorded three neurological deficits [21]. Elsherif et al. reported 33 patients with high-energy trauma, of whom 9 presented incomplete neurological lesions and 24 complete lesions, representing one of the highest proportions of neurological impairment across published cohorts [16]. Aleissa et al., in a smaller cohort of nine patients aged between 14 and 60 years, documented six cases of partial peripheral neurological deficit [14]. Vankipuram et al. analyzed six patients (mean age 37.5 years, range 19–61), reporting neurological impairment in three cases, while the remaining three had no deficits [10]. Chou et al. reported six neurological deficits among 12 patients (mean age 40 years, range 18–78) [27], while Pearson et al. described three cases of cauda equina syndrome in a cohort of 31 patients [28]. Earlier studies also provided important insights. Lindahl presented 36 patients (mean age 30.5 years, range 15–66), of whom 29 suffered from cauda equina syndrome and 1 from paraplegia [32]. Ayoub, in a series of 28 patients with a mean age of 33.7 years, documented 11 cases of complete cauda equina syndrome and 17 cases of incomplete neurological impairment [34]. Markel, in a single case report of a 26-year-old patient, described a motor deficit of the left lower limb [35]. Overall, the analysis highlights that neurological involvement is present in nearly half of patients with spinopelvic dissociation, with cauda equina syndrome representing the most severe and frequent manifestation in larger cohorts. The variability in reported deficits—from isolated radicular pain to complete paraplegia—reflects both the heterogeneity of fracture morphology and the severity of displacement. Diagnostic work-up in all modern series relied primarily on computed tomography (CT), which is the principal imaging modality for delineating fracture morphology and detecting displacement. Magnetic resonance imaging (MRI) was occasionally employed in selected cases to assess nerve root compression or to investigate soft tissue and ligamentous injuries. However, the literature also underscores the frequent diagnostic delay, particularly in polytraumatized patients presenting with altered consciousness or competing life-threatening injuries.

### 4.4. Management Strategies

Surgical fixation was the most frequently reported treatment in modern series, whereas nonoperative management was generally reserved for selected non-displaced fractures or patients considered unfit for surgery. Timing of surgery was reported heterogeneously across the literature but provides important insights into current practice. Taylor et al., in their series of 48 patients, reported a mean time to surgery of 4.8 days (range 0–22) [8]. Young et al. described a mean delay of 5.2 days (range 1–19) for 40 patients treated with open lumbopelvic fixation, whereas in their subsequent cohort of 53 patients managed percutaneously, the mean interval was 3.6 days (range 0–14) [11,15]. Shi et al., in the largest available series of 138 patients, reported an average delay of 8.6 days (range 0–43.5) [17]. Tian et al., analyzing 28 patients, documented a mean time to definitive fixation of 5.9 days (range 2–15) [21]. Elsherif et al., in 33 patients, reported an average of 6.2 days (range 2–21) [16]. Lindahl similarly observed a mean surgical delay of 7 days (range 0–25) in 36 patients [32]. With regard to surgical strategies, open reduction and internal fixation with triangular lumbopelvic constructs were predominantly employed in earlier cohorts, particularly in patients with severe displacement or associated neurological deficits, as described by Lindahl (2014) and Ayoub (2012) [32,34]. In more recent studies, percutaneous fixation techniques have been increasingly adopted, especially in patients without severe neurological impairment, and have been associated with reduced blood loss, shorter operative times, and earlier mobilization, as reported by Young (2023) and Taylor (2025) [8,15]. Cases presenting with cauda equina syndrome often underwent open decompression combined with fixation, as in Taylor (2025) and Elsherif (2023) [8,16]. Taken together, these findings suggest that surgical stabilization is commonly undertaken during the early post-injury period when the patient’s overall condition permits, while the choice between open and percutaneous approaches is primarily influenced by fracture morphology, displacement, neurological compromise, and the need for direct decompression.

### 4.5. Neurological and Functional Outcomes

Across the 29 published studies and the present case series, comprising 30 studies/series and 739 patients overall, outcome reporting was heterogeneous, but several cohorts provided numerically explicit results that allow for a precise synthesis. In the present case series (two patients), one patient achieved full recovery, whereas one had a residual urinary urgency at follow-up. Esteves et al. (single pediatric case, 5 years) documented full recovery [8]. Vankipuram et al. (six patients; mean age 37.5 years, 19–61) reported one persistent bladder dysfunction and one ASIA A status at follow-up, indicating substantial residual morbidity in part of the cohort [10]. Young et al. (2024; 40 patients; mean age 40 years, 16–73) specified the evolution of sphincter deficits; among those presenting with bowel/bladder dysfunction (*n* = 12), nine achieved complete recovery [11]. The same series also included patients with isolated sensory (*n* = 3) and motor (*n* = 5) deficits at presentation, underscoring the heterogeneity of neurological impairment and recuperation [11]. In a subsequent cohort, Young et al. (2023; 53 patients managed percutaneously) explicitly noted neurologic recovery while also emphasizing in their discussion that paresthesias were the most common long-term neurologic sequela; numerators were not provided in the table for that series [15]. Aleissa et al. (2023; nine patients, 14–60 years) reported complete neurological improvements in all nine patients [14]. Luo et al. (2023; 14 patients; mean age 30.3 years, 13–72) described a good rate of pelvic function and neurological recovery, without detailing numerators in the extracted table [18]. Tian et al. (2021; 28 patients; mean age 30 years, 16–59) documented three partial neurological recoveries, in line with the three neurological deficits reported at presentation for that cohort [21]. Jindal et al. (2022; 22 patients) stated full recovery of neurological deficits, although the exact numerator at risk was not specified in the table [25]. Miyamoto et al. (2020; six patients) recorded four cases with moderate disability at follow-up, indicating a relatively high rate of functional limitation in that small series [26]. Xie et al. (2018; 15 patients) provided one of the more granular breakdowns: three patients achieved full recovery, five had isolated paresthesia as the only residual symptom, and four had persistent combined deficits including paresthesia with lower-extremity involvement and bladder dysfunction; the remainder were not fully specified in the table, but the distribution clearly shows mixed but frequently favorable trajectories [1]. Two larger historical cohorts offer additional clarity on neurological endpoints. Lindahl (2014; 36 patients; mean age 30.5 years, 15–66) reported that 17 of 29 patients (59%) presenting with cauda equina syndrome (Gibbons grade 4) had full recovery of bladder and bowel function; importantly, twelve of the 36 patients (33%) had permanent bladder and/or bowel dysfunction at final follow-up, highlighting the substantial risk of enduring sphincter impairment even with definitive fixation [32]. Park et al. (2012; 71 patients) noted that neither of two documented cases (subgroup explicitly discussed in the outcome field) showed neurological recovery, a finding that, while limited to those specific patients, underscores the potential for poor outcomes in severe presentations [33]. Ayoub (2012; 28 patients; mean age 33.7 years) observed universal neurological improvement, with 11 complete and 17 incomplete recoveries within the cohort [34]. Finally, Markel (1993; single case, 26 years) reported full restoration of limb function at two years, illustrating the possibility of excellent long-term recovery even after profound initial deficits in isolated cases [35]. The reporting of complications was inconsistent across studies, and explicit implant-related complication numerators were rarely tabulated in the extracted fields. Where qualitative statements were provided, long-term paresthesias appeared as the most frequent residual complaint after percutaneous stabilization in selected cohorts (Young et al., 2023) [15]. The Lindahl series (2014) furnishes the most concrete sphincteric endpoint, with 33% of the entire cohort retaining permanent bladder and/or bowel dysfunction at final follow-up despite modern fixation strategies, aligning with the notion that initial cauda equina syndrome is a strong predictor of long-term neurogenic sequelae [32]. Overall, among studies that supplied explicit numerators, the spectrum of outcomes ranged from complete neurological recovery (e.g., Aleissa, 9/9; multiple single-case reports) to persistent moderate disability (e.g., Miyamoto, 4/6) and permanent sphincter dysfunction in a non-trivial proportion (e.g., Lindahl, 12/36), reflecting the combined influence of fracture morphology, initial neurological status, and timeliness and type of surgical stabilization [14,26,32].

## 5. Discussion

Traumatic sacral fracture–dislocations with spinopelvic dissociation are uncommon but clinically demanding injuries of the lumbopelvic junction. Although they account for only a small proportion of sacral fractures, they may be associated with neurological compromise, multisystem trauma, and long-term functional limitations [5,36]. The two adolescent cases presented in this report add age-specific clinical information to a literature that predominantly concerns adult or mixed-age populations. More importantly, they illustrate that similar diagnostic labels may encompass markedly different fracture morphologies, neurological risks, and surgical requirements.

The findings of the systematic review should be interpreted in the context of this clinical heterogeneity. Spinopelvic dissociation most frequently follows high-energy trauma, particularly falls from height and motor-vehicle crashes, and is often accompanied by associated injuries that may delay diagnosis and definitive treatment. The majority of patients described in the literature are young adults sustaining high-energy trauma, most frequently road-traffic accidents or falls from height [11,19]. Neurological deficits are common, although their reported frequency varies across series, supporting careful neurological assessment and timely multidisciplinary evaluation [5,37]. Delayed diagnosis remains a relevant concern, particularly in polytraumatized patients with competing life-threatening injuries or complex fracture morphology [4,32]. The recent literature has increasingly described minimally invasive percutaneous strategies, which may reduce operative time, blood loss, and wound morbidity in appropriately selected patients [11,22]. However, this apparent advantage cannot be interpreted as evidence of superiority because open reduction and decompression are more often selected for patients with greater displacement, canal compromise, or neurological compression [38]. The different outcomes observed in our two cases are consistent with this selection pattern: the patient treated percutaneously had a fracture amenable to closed reduction and no sphincter dysfunction, whereas the patient requiring open decompression had severe comminution, S1–S2 spondyloptosis, foraminal involvement, and a sacral root lesion. Her residual urinary symptoms, therefore, appear more plausibly related to the severity of the initial neurological injury than to the open approach itself. Intraoperative navigation may assist screw placement and reduce radiation exposure, but the magnitude of its clinical benefit remains to be established in larger comparative studies [39,40].

When considered alongside the reviewed evidence, the two adolescent cases reflect the principal outcome patterns identified in the literature. The complete neurological recovery observed after percutaneous fixation in Case 1 is consistent with reports of favorable recovery in selected patients with reducible fractures and limited initial neurological impairment. Conversely, the persistent urinary urgency in Case 2 parallels the residual sphincter dysfunction reported in patients with cauda equina involvement, marked displacement, or severe sacral comminution. These contrasting courses therefore support the interpretation that neurological recovery is influenced primarily by the severity and reversibility of the initial neural injury, whereas the surgical approach is selected according to fracture morphology and the need for direct decompression. The cases should consequently be viewed as complementary clinical examples rather than as a direct comparison of open and percutaneous fixation.

### 5.1. Classification

The classification of sacral fractures has evolved considerably over the past decades, reflecting a progressive effort to link fracture morphology with prognosis and therapeutic decision-making. The most widely adopted and historically influential system is that of Denis, which categorizes sacral fractures according to the coronal plane and their relationship with the neural foramina. Denis type I fractures are located lateral to the foramina and are associated with a relatively low incidence of neurological injury, estimated at approximately 6%. Type II fractures cross the foramina and carry a significantly higher risk of neurological compromise, up to 28%. Type III fractures are medial to the foramina and involve the central sacral canal, with an incidence of neurological injury approaching 56% [36,41]. The Roy-Camille classification, later modified by Strange-Vognsen and Lebech, shifted the focus from the coronal to the sagittal plane, emphasizing the cranio-caudal displacement of the fracture fragments. Four types are described: type I with isolated kyphotic angulation of the cranial fragment; type II with kyphosis and associated retrolisthesis; type III with complete anterolisthesis; and type IV with comminution of the S1 vertebral body [42]. This classification provides useful insights into the stability and mechanical consequences of these injuries, particularly in high-energy trauma. Another important contribution was made by Isler, who introduced a classification specifically addressing longitudinal fractures corresponding to Denis type II. This system considers the relationship of the fracture line with the L5–S1 facet joint, distinguishing between lateral (type I), transarticular (type II), and medial (type III) trajectories. The medial variant is considered inherently unstable, as it compromises both the osseous and ligamentous structures stabilizing the lumbosacral junction [43]. In recent years, attempts have been made to unify and refine classification schemes. The AO Spine Sacral Injury Classification System was developed to integrate existing models into a more comprehensive framework, thereby facilitating communication across multidisciplinary teams and enabling more consistent treatment algorithms. This system incorporates four main elements: fracture morphology, integrity of the posterior ligamentous complex, neurological status, and clinical modifiers. From a morphological perspective, type A injuries involve low sacrococcygeal fractures without posterior instability, type B include unilateral vertical fractures with posterior pelvic instability but preserved spinopelvic stability, and type C encompass bilateral vertical fractures, U-shaped sacral fractures, and injuries involving the L5–S1 facet joints, all of which are characterized by spinopelvic instability [44]. More recently, Shi and colleagues proposed the 301 Spinopelvic Dissociation Classification, which extends the definition of spinopelvic dissociation to include sacroiliac joint involvement. This novel system stratifies injuries into three groups: group I, trans-sacral fractures without sacroiliac involvement; group II, unilateral trans-sacral fracture with contralateral sacroiliac injury; and group III, bilateral sacroiliac joint injuries with increasing complexity. While the 301 SPD classification provides a potentially useful therapeutic framework, its validation remains limited, as the original study included only 30 patients with a relatively short follow-up of 21.9 months [17]. Taken together, these classification systems highlight the complexity of sacral fractures and their associated instability patterns. Our review confirms that the Denis and Roy-Camille classifications remain the most commonly applied in clinical reports, although newer systems such as the AO Spine and 301 SPD classifications have been gaining attention in recent publications. Nevertheless, no single system has yet achieved universal adoption, underscoring the ongoing challenge of linking radiological morphology to prognosis and treatment strategies.

### 5.2. Decision-Making

Traumatic lumbosacral instability (TLSI) and its associated injury patterns are typically the result of high-energy trauma and are frequently accompanied by multiple concomitant injuries. In this context, early decision-making benefits from a multidisciplinary approach, as the initial priorities may include life-saving procedures addressing hemodynamic instability, thoracoabdominal injuries, or intracranial trauma [5,45,46]. The anatomical complexity of the lumbopelvic junction and the dual involvement of both spine and pelvis also present organizational challenges. In some trauma centers, the treating surgeon may have expertise in both spinal and pelvic trauma, allowing for comprehensive management of these cases. However, in many institutions, spine surgeons and pelvic trauma surgeons work separately, which can create gaps in expertise. For example, pelvic surgeons may be less experienced in lumbosacral stabilization and decompression, whereas spine surgeons may not be familiar with pelvic ring fixation techniques. In such situations, close and coordinated collaboration between the two teams may help optimize treatment planning and avoid unnecessary delays [39,47]. Several clinical and radiological factors influence decision-making and the timing of surgery. These include the patient’s physiological status, the severity of neurological deficits, the degree of displacement and instability, and the presence of extensive soft tissue compromise in the lumbosacral and gluteal regions. Lindahl and colleagues identified neurological involvement and fracture displacement as important predictors of outcome in spinopelvic dissociation, supporting early recognition and individualized stabilization strategies [32]. To address the heterogeneity of these injuries and aid treatment planning, Lehman and colleagues introduced the Lumbosacral Injury Classification System (LSICS). This system evaluates three primary injury domains: fracture morphology, posterior ligamentous complex integrity, and neurological status. In addition, three clinical modifiers are incorporated: the systemic injury burden and physiological status of the polytraumatized patient, the soft tissue condition, and the anticipated time to mobilization. LSICS therefore offers a structured framework for decision-making in complex lumbosacral injuries, including lumbosacral dislocation, spinopelvic dissociation, and high-energy vertically unstable sacral fractures [48]. Although LSICS may be useful in clinical practice, its application should remain contextualized within the broader clinical scenario, particularly in polytraumatized patients, where timing and sequencing of interventions may be dictated by life-threatening injuries rather than fracture morphology alone.

### 5.3. Treatment

Surgical treatment is frequently used for sacral fracture–dislocations and spinopelvic dissociation (SPDI), whereas nonoperative management may be considered in carefully selected situations. These may include minimally displaced high transverse sacral fractures (<10 mm), fractures distal to the sacroiliac joint, patients with substantial comorbidities or high anesthetic risk, or those unlikely to ambulate for at least 2–3 months after trauma. Even in these circumstances, close follow-up is advisable because nonoperative management has been associated with progressive deformity, sagittal imbalance, chronic pain, malunion, and potential neurological deterioration [36,48]. The goals of surgery are to decompress neural elements when indicated, restore alignment, and re-establish lumbosacral and posterior pelvic stability. Stable fixation may facilitate earlier mobilization and weight-bearing, potentially reducing complications in polytraumatized patients. Timing is influenced by neurological status, fracture morphology, associated injuries, and overall physiological condition. Although some reports associate intervention within 48 h to 2 weeks with favorable neurological and functional outcomes, the optimal timing remains uncertain, and definitive fixation is often delayed by concomitant life-threatening injuries. Two large systematic reviews reported mean times to surgery of 5 days (range 0–45) and 8.6 days (range 0–43.5), despite neurological deficits being present in 80% and 68% of cases [5,49]. Triangular lumbopelvic fixation (TLPF) and lumbopelvic fixation (LPF) are commonly reported constructs for SPDI. Bäcker et al. found that TLPF and LPF were employed in 69% of cases and may provide greater biomechanical stability than sacral screw fixation alone, with the potential to support earlier mobilization and weight-bearing [5]. Biomechanical studies also suggest advantages of triangular constructs. Schildhauer and colleagues reported that combining vertical bilateral rods with a transverse sacral screw increased resistance to flexion and limited caudal migration of the cranial sacrum [38]. Peng and colleagues, using finite element modeling, found lower stability with trans-sacral–transiliac screw fixation than with TLPF and bilateral triangular constructs, particularly in U- and H-type sacral fractures with traumatic spinopelvic dissociation [50]. Lindahl et al. described a construct for H-shaped spinopelvic dissociation consisting of bilateral lumbar pedicle screws in L4 and L5, longitudinal rods, transverse connectors, and bilateral iliac screws. This configuration may permit simultaneous or stepwise reduction in the cranial and lateral hemipelves, correction of sagittal and rotational deformity, and realignment of the caudal fragment [31]. Alternative constructs, such as bilateral iliolumbar instrumentation without horizontal fixation, have been used in selected cases, although available biomechanical and clinical data suggest lower stability. Shetty et al. reported favorable outcomes with percutaneous sacroiliac screw fixation combined with lumbopelvic fixation, whereas constructs lacking transverse elements appeared less effective in controlling coronal displacement [51]. Jazini et al. similarly found in a cadaveric model that lumbopelvic fixation increased stability compared with iliosacral screws [52]. Sevillano-Perez et al. suggested that routine extension of fixation to L4 may not be necessary except in severe deformity or when sagittal correction cannot otherwise be achieved, and highlighted a possible adjunctive role for transilio-trans-sacral fixation [40]. Overall, these findings support selecting fixation constructs according to the direction and magnitude of instability, while recognizing that current clinical evidence does not establish the superiority of one surgical strategy over another [39].

### 5.4. Limitations and Future Directions

Despite the growing number of publications on sacral fractures with spinopelvic dissociation, the available evidence remains limited by several important constraints. Most studies are retrospective case series or case reports, with substantial heterogeneity in patient populations, fracture patterns, and treatment strategies. Randomized controlled trials and large prospective cohorts are lacking, which limits the strength of recommendations that can be drawn for clinical decision-making [5]. Another limitation is the variability in classification systems employed across studies. While the Denis and Roy-Camille systems are most frequently used, more recent proposals such as the AO Spine classification and the 301 SPD classification have not yet achieved widespread clinical adoption. This inconsistency hampers comparison between studies and complicates the development of standardized treatment algorithms. Outcome reporting is also inconsistent, with studies using different functional scales, radiographic criteria, and neurological assessments. Follow-up durations are often short, and long-term data on functional recovery, quality of life, and chronic pain remain scarce. Moreover, complications such as infection, hardware failure, or malunion are not uniformly reported, further limiting the generalizability of current findings [49]. A further limitation is confounding by indication: open fixation and decompression are generally selected for more severe injuries, whereas percutaneous approaches are more often used in less displaced fractures without a clear need for direct neural decompression. Consequently, unadjusted differences in outcomes cannot be attributed to the surgical approach alone. This limitation is illustrated by our two cases, in which the poorer neurological outcome occurred in the patient with the more severe initial fracture and root injury. Future research should prioritize multicenter prospective registries with standardized definitions of fracture severity, neurological status, surgical indications, complications, and long-term outcomes. Such studies may provide more robust evidence regarding timing, fixation strategy, and patient selection. Consensus initiatives involving spine surgeons, pelvic trauma surgeons, and multidisciplinary trauma teams may also help harmonize reporting and treatment pathways. Minimally invasive and navigation-assisted techniques remain promising, but their comparative benefits require validation in larger studies that account for baseline injury severity.

The methodological appraisal reinforces the need for cautious interpretation. The available literature is dominated by small retrospective series, and only a limited number of studies directly compared treatment strategies. Even in comparative cohorts, treatment allocation was determined by clinical indication rather than randomization, creating substantial confounding by injury severity and neurological status. Therefore, the apparent perioperative advantages associated with percutaneous fixation cannot be separated confidently from differences in baseline case complexity.

## 6. Conclusions

Sacral fracture–dislocations with spinopelvic dissociation are rare and heterogeneous injuries that may be associated with neurological compromise and multisystem trauma. Our two adolescent cases illustrate that both percutaneous fixation and open reduction with decompression can provide satisfactory mechanical stabilization when selected according to fracture morphology, displacement, neurological status, and the need for direct decompression. The different neurological outcomes observed in the two patients likely reflect differences in initial injury severity, including the presence of severe comminution, spondyloptosis, foraminal involvement, and sacral root injury, rather than the superiority or inferiority of either surgical approach. Early stabilization may be beneficial when the patient’s clinical condition permits, but the optimal timing remains uncertain. Percutaneous techniques may reduce surgical morbidity in appropriately selected patients, whereas open approaches may be required when direct reduction or neural decompression is necessary. Intraoperative navigation may support accurate implant placement, although its effect on long-term clinical outcomes requires further study. Because the available evidence is predominantly retrospective, heterogeneous, and affected by treatment-selection bias, no firm comparison between open and percutaneous fixation can currently be made. Multicenter prospective studies using standardized neurological and functional outcomes are needed to clarify indications and support evidence-based surgical decision-making.

## Figures and Tables

**Table 1:** Demographic data of patients and systematic review.

Author	Year	N° Patients	Sex	Age	Neurological Injury	Mechanism of Injury	Timing	Surgical Treatment	Outcome
**Present study** **.**	2025	2	F	15.5 (14–17)	1 hypoesthesia left lower limb	1 motor-vehicle accident; 1 fall from height	48 h	Pelvic fixation	One full recovery, one residual of urinary incontinence
**Taylor et al.** [8]	2025	48	20 F 28 M	49.2 (open group) 42.6 (perc. Group)	3 cauda open; 4 cauda percutaneous	-	-	21 open 27 percutaneous	-
**Esteves et al.** [9]	2024	1	M	5	-	-	-	Open reduction	Full recovery
**Vankipuram et al.** [10]	2023	6	4 M 2 F	37.5 (19–61)	3 without deficits; 3 motor deficits	-	-	Reduction and stabilization using dual triangular osteosynthesis constructs	1 bladder disfunction, 1 ASIA A
**Young et al.** [11]	2024	40	21 M 19 F	40 (16–73)	3 sensorial deficits; 5 motor deficits; 12 bladder deficits	20 motor-vehicle accidents; 9 falls from height; 2 crushes; 9 other	-	Open lumbopelvic fixation	9 full recovery of sphincter deficits
**Etebari et al.** [12]	2023	1	M	74	Yes	Chiropractic adjustment	-	Conservative	Mild residual neuropathy
**Altwaijri et al.** [13]	2023	1	M	32	Loss of power in L3-S1	Fall from height	-	Spinopelvic fixation and fusion with rigid fixation done	Full recovery
**Aleissa et al.** [14]	2023	9	7 M 2 F	28 (14–60)	6 partial peripheral deficit	4 motor-vehicle accidents; 1 fall from height; 1 due to seizure	-	-	Complete neurological improvements
**Young et al.** [15]	2023	53	-	-	15 neurological deficits (radicular pain and paresthesias)	-	-	Percutaneous iliosacral screw fixation was use	Neurologic recovery
**Elsherif et al.** [16]	2023	33	9 F 24 M	30	9 incomplete neurological injuries; 24 complete neurological injuries	21 falls from height; 12 motor-vehicle accidents	48 h	18 direct decompressions 15 indirect decompressions	-
**Shi et al.** [18]	2023	138	20 M 10 F	40.7 (16–68)	9 nerve injuries	12 falls from height; 9 motor-vehicle accidents; 9 compressions	12 day	Direct decompression and open reduction	-
**Luo et al.** [18]	2022	14	4 M 10 F	30.3 (13–72)	-	-	-	Bilateral S2AI screws	Good rate of pelvic function and neurological recovery
**Obey et al.** [20]	2021	82		56.8	-	-	-	-	-
**Fernández et al.** [21]	2021	11		34 (13–59)	-	-	-	-	-
**Tian et al.** [21]	2021	28	16 M 12 F	30 (16–59)	3 neurological deficits	19 motor-vehicle accidents; 9 falls from height	-	-	3 partial recoveries
**Erkan et al.** [22]	2020	19		47.2 (38–55)	-	-	-	-	-
**Lee et al.** [23]	2020	2	1 F	20	-	Fall from height	-	-	-
**Liu et al.** [24]	2020	Q		35 (24–46)	-	-	-	-	-
**Jindal et al.** [25]	2020	22	-	36.6 (17–62)	-	18 road-traffic accidents; 4 falls from height	9.7 day (3–21)	-	Full recovery deficit
**Miyamoto et al.** [26]	2020	6	2 M 6 F	29 (20–48)	-	3 MVA; 3 falls from height	-	-	4 moderate disability
**Chou et al.** [27]	2018	12	-	40 (18–78)	6 neurological deficits	-	-	-	-
**Xie et al.** [1]	2018	15	9 M 6 F	28.8 (15–55)	-	Fall from height	-	-	4 paresthesia, lower extremity motor deficit and bladder dysfunction; 3 lower deficit and bladder dysfunction; 3 full recovery; 5 only paresthesia
**Pearson et al.** [28]	2018	31	20 M 11 F	41	3 cauda	-	-	15 open reduction; 16 closed reduction	-
**Tian et al.** [29]	2018	18	14 M 4 F	33.1 (18–55)	-	16 falls from height; 2 traffic accidents	5–35 days	-	8 reduced paresthesia; 1 partial dysfunction of the bladder; 2 not have completely restored unilateral plantar flexion of the ankle
**Kanezaki et al.** [30]	2018	10	6 M 3 F	50 (27–78)	-	-	-	Minimally invasive triangular osteosynthesis	-
**Yazdi et al.** [31]	2015	1	1 M	16	None	Fall from height	Open reduction and internal fixation	-	-
**Lindahl et al.** [32]	2014	36	18 M 18 F	30.5 (15–66)	29 cauda; 1 paraplegia	-	21 days	Open reduction and internal fixation	Seventeen of 29 patients with a cauda equina injury (Gibbons grade 4) had full recovery of their bladder and bowel functions. Of 36 patients, seven underwent a full neurological recovery. Eight patients were left with sensory deficits, while nine patients had motor and sensory deficits in the lower extremities. Twelve patients had permanent bladder and/or bowel dysfunction.
**Park et al.** [33]	2012	71	32 M 39 F	43.1 (19–91)	-	3 falls from height; 13 accident falls from height; 35 traffic accidents; 12 slips; 5 begin buried	-	Conservative treatment for all non-displaced fracture; percutaneous iliosacral screw fixation was used in displaced fracture	Neither patient showed any neurologic recovery.
**Ayoub et al.** [34]	2012	28	17 M 11 F	33.7	11 complete cauda; 17 incomplete cauda	11 falls from height	9 motorcycle accidents; 8 pedestrian accidents	14 indirect decompression + percutaneous fixation; 14 direct decompression + open fixation	17 incomplete neurological improvement; 11 complete neurological improvement
**Markel et al.** [35]	1993	1	1 M	26	Motor deficit of left lower limb	Traffic accident	-	Open reduction and internal fixation with only screw and plate	Full recovery at 2 years FU

## Data Availability

The original contributions presented in this study are included in the article. Further inquiries can be directed to the corresponding author.

## Supplementary Materials

The following supporting information can be downloaded at https://www.mdpi.com/article/10.3390/jcm15134914/s1, Table S1: PRISMA 2020 Checklist [6,8]; Table S2: JBI Critical Appraisal of Included Studies [9,10,11,12,13,14,15,16,17,18,19,20,21,22,23,24,25,26,27,28,29,30,31,32,33,34,35,36].
