# Supplementary material for: Open and Percutaneous Fixation of Traumatic Sacral Fracture–Dislocation with Spinopelvic Dissociation: Two Adolescent Cases and a Systematic Literature Review
Source: J Clin Med. 2026 Jun 24;15(13):4914. doi: 10.3390/jcm15134914 (PMC13361836; doi:10.3390/jcm15134914)
Supplement: Supplementary file 1 [file jcm-15-04914-s001.zip › jcm-4368254-supplementary.pdf]

PRISMA 2020 Checklist

Supplementary Table S1. PRISMA 2020 Checklist

Section and Topic	Item #	Checklist item	Location where item is reported
**TITLE**			
Title	1	Identify the report as a systematic review.	Title, p. 1
**ABSTRACT**			
Abstract	2	See the PRISMA 2020 for Abstracts checklist.	Abstract, pp. 1-2
**INTRODUCTION**			
Rationale	3	Describe the rationale for the review in the context of existing knowledge.	Introduction, p. 2
Objectives	4	Provide an explicit statement of the objective(s) or question(s) the review addresses.	End of Introduction, p. 2
**METHODS**			
Eligibility criteria	5	Specify the inclusion and exclusion criteria for the review and how studies were grouped for the syntheses.	Section 2.2, p. 4; synthesis groupings in Sections 4.1-4.4, pp. 9-12
Information sources	6	Specify all databases, registers, websites, organisations, reference lists and other sources searched or consulted to identify studies. Specify the date when each source was last searched or consulted.	Section 2.1, pp. 2-3 (PubMed/MEDLINE, Scopus, Web of Science, reference lists; final search 30 August 2025)
Search strategy	7	Present the full search strategies for all databases, registers and websites, including any filters and limits used.	Section 2.1, pp. 2-3
Selection process	8	Specify the methods used to decide whether a study met the inclusion criteria of the review, including how many reviewers screened each record and each report retrieved, whether they worked independently, and if applicable, details of automation tools used in the process.	Section 2.3, p. 4; Figure 1
Data collection process	9	Specify the methods used to collect data from reports, including how many reviewers collected data from each report, whether they worked independently, any processes for obtaining or confirming data from study investigators, and if applicable, details of automation tools used in the process.	Section 2.3, p. 4 (variables and data-collection process; reviewer independence for extraction not explicitly stated)
Data items	10a	List and define all outcomes for which data were sought. Specify whether all results that were compatible with each outcome domain in each study were sought (e.g. for all measures, time points, analyses), and if not, the methods used to decide which results to collect.	Section 2.3, p. 4; Sections 4.1-4.4, pp. 9-12
	10b	List and define all other variables for which data were sought (e.g. participant and intervention characteristics, funding sources). Describe any assumptions made about any missing or unclear information.	Section 2.3, p. 4
Study risk of bias assessment	11	Specify the methods used to assess risk of bias in the included studies, including details of the tool(s) used, how many reviewers assessed each study and whether they worked independently, and if applicable, details of automation tools used in the process.	Section 2.5, p. 5; Supplementary Table S2
Effect measures	12	Specify for each outcome the effect measure(s) (e.g. risk ratio, mean difference) used in the synthesis or presentation of results.	Section 2.4, pp. 4-5 (counts, percentages, means, ranges, and standard deviations; no meta-analysis)
Synthesis methods	13a	Describe the processes used to decide which studies were eligible for each synthesis (e.g. tabulating the study intervention characteristics and comparing against the planned groups for each synthesis (item #5)).	Sections 2.2-2.4, pp. 4-5; Sections

Section and Topic	Item #	Checklist item	Location where item is reported
			4.1-4.4, pp. 9-12
	13b	Describe any methods required to prepare the data for presentation or synthesis, such as handling of missing summary statistics, or data conversions.	Sections 2.3-2.4, pp. 4-5 (no imputations or statistical conversions reported)
	13c	Describe any methods used to tabulate or visually display results of individual studies and syntheses.	Figure 1; Table 1; Sections 4.1-4.4, pp. 9-12
	13d	Describe any methods used to synthesize results and provide a rationale for the choice(s). If meta-analysis was performed, describe the model(s), method(s) to identify the presence and extent of statistical heterogeneity, and software package(s) used.	Section 2.4, pp. 4-5
	13e	Describe any methods used to explore possible causes of heterogeneity among study results (e.g. subgroup analysis, meta-regression).	Section 2.4, pp. 4-5; Discussion and Section 5.4, pp. 12-16 (qualitative exploration only)
	13f	Describe any sensitivity analyses conducted to assess robustness of the synthesized results.	Not applicable - no sensitivity analysis was conducted because no meta-analysis was performed
Reporting bias assessment	14	Describe any methods used to assess risk of bias due to missing results in a synthesis (arising from reporting biases).	Not formally assessed because the evidence base was predominantly small, heterogeneous case reports and observational series and no meta-analysis was performed
Certainty assessment	15	Describe any methods used to assess certainty (or confidence) in the body of evidence for an outcome.	Not formally graded; methodological quality was assessed with design-specific JBI tools (Section 2.5, p. 5; Supplementary Table S2)
**RESULTS**			
Study selection	16a	Describe the results of the search and selection process, from the number of records identified in the search to the number of studies included in the review, ideally using a flow diagram.	Section 2.3, p. 4; Figure 1
	16b	Cite studies that might appear to meet the inclusion criteria, but which were excluded, and explain why they were excluded.	Section 2.3, p. 4; Figure 1 (numbers and reasons for full-text exclusions are reported; no citation-level

Section and Topic	Item #	Checklist item	Location where item is reported
			exclusion list)
Study characteristics	17	Cite each included study and present its characteristics.	Table 1; Sections 4.1-4.4, pp. 9-12; references [1,11-38]
Risk of bias in studies	18	Present assessments of risk of bias for each included study.	Methodological Quality of the Included Studies, pp. 8-9; Supplementary Table S2
Results of individual studies	19	For all outcomes, present, for each study: (a) summary statistics for each group (where appropriate) and (b) an effect estimate and its precision (e.g. confidence/credible interval), ideally using structured tables or plots.	Table 1; Sections 4.1-4.4, pp. 9-12 (narrative study-level results where reported)
Results of syntheses	20a	For each synthesis, briefly summarise the characteristics and risk of bias among contributing studies.	Methodological Quality of the Included Studies, pp. 8-9; Sections 4.1-4.4, pp. 9-12; Supplementary Table S2
	20b	Present results of all statistical syntheses conducted. If meta-analysis was done, present for each the summary estimate and its precision (e.g. confidence/credible interval) and measures of statistical heterogeneity. If comparing groups, describe the direction of the effect.	Not applicable - no statistical synthesis or meta-analysis was conducted; narrative synthesis in Sections 4.1-4.4, pp. 9-12
	20c	Present results of all investigations of possible causes of heterogeneity among study results.	Sections 4.1-4.4, pp. 9-12; Discussion, pp. 12-16 (qualitative assessment)
	20d	Present results of all sensitivity analyses conducted to assess the robustness of the synthesized results.	Not applicable - no sensitivity analyses were conducted
Reporting biases	21	Present assessments of risk of bias due to missing results (arising from reporting biases) for each synthesis assessed.	Not formally assessed because of the small, heterogeneous, predominantly non-comparative evidence base
Certainty of evidence	22	Present assessments of certainty (or confidence) in the body of evidence for each outcome assessed.	Not formally graded; confidence in comparative conclusions is discussed in Section 5.4 and the Conclusion, pp. 15-16
**DISCUSSION**			

PRISMA 2020 Checklist

Section and Topic	Item #	Checklist item	Location where item is reported
Discussion	23a	Provide a general interpretation of the results in the context of other evidence.	Discussion, pp. 12-16
	23b	Discuss any limitations of the evidence included in the review.	Section 5.4, pp. 15-16
	23c	Discuss any limitations of the review processes used.	Section 5.4, pp. 15-16 (heterogeneity, retrospective designs, inconsistent reporting, limited follow-up, and confounding; review-process limitations are not separately detailed)
	23d	Discuss implications of the results for practice, policy, and future research.	Discussion, Section 5.4, and Conclusion, pp. 12-16
**OTHER INFORMATION**			
Registration and protocol	24a	Provide registration information for the review, including register name and registration number, or state that the review was not registered.	Section 2.1, p. 3 (review not prospectively registered)
	24b	Indicate where the review protocol can be accessed, or state that a protocol was not prepared.	Section 2.1, p. 3 (no formal protocol was prepared or published)
	24c	Describe and explain any amendments to information provided at registration or in the protocol.	Not applicable - no registered or published protocol was prepared
Support	25	Describe sources of financial or non-financial support for the review, and the role of the funders or sponsors in the review.	Funding statement, p. 17 (no external funding)
Competing interests	26	Declare any competing interests of review authors.	Conflicts of Interest statement, p. 17
Availability of data, code and other materials	27	Report which of the following are publicly available and where they can be found: template data collection forms; data extracted from included studies; data used for all analyses; analytic code; any other materials used in the review.	Data Availability Statement, p. 17; Supplementary Table S2 and this PRISMA 2020 checklist

Manuscript: Open and Percutaneous Fixation of Traumatic Sacral Fracture-Dislocation with Spinopelvic Dissociation: Two Adolescent Cases and a Systematic Literature Review

From: Page MJ, McKenzie JE, Bossuyt PM, Boutron I, Hoffmann TC, Mulrow CD, et al. The PRISMA 2020 statement: an updated guideline for reporting systematic reviews. BMJ 2021;372:n71. doi: 10.1136/bmj.n71. This work is licensed under CC BY 4.0. To view a copy of this license, visit https://creativecommons.org/licenses/by/4.0/

Supplementary Table S2. JBI Critical Appraisal of Included Studies

Code	Meaning
Y	Yes
N	No
U	Unclear
NA	Not applicable

Overall concern is an author-defined synthesis informed by the item-level JBI assessment. No study was excluded solely on methodological quality. Where reporting was insufficient, the item was rated U (Unclear). A second author should independently verify all judgments before submission.

A. JBI Critical Appraisal Checklist for Case Reports (8 items)

Study	Year	Q1	Q2	Q3	Q4	Q5	Q6	Q7	Q8	Overall concern	Notes
Esteves et al.	2024	Y	Y	Y	Y	Y	Y	U	Y	Low concern	Detailed presentation, imaging, treatment and serial follow-up; adverse-event reporting not fully explicit.
Etebari et al.	2023	Y	Y	Y	Y	Y	Y	U	Y	Moderate concern	Clinical course is described, but follow-up duration and adverse-event reporting are limited.
Altwaijri et al.	2023	Y	Y	Y	Y	Y	Y	U	Y	Moderate concern	Diagnostic and therapeutic details are reported; long-term follow-up and adverse-event reporting remain limited.
Safaie Yazdi et al.	2015	Y	Y	Y	Y	Y	Y	U	Y	Moderate concern	Adequate diagnostic and treatment description, with limited long-term and adverse-event reporting.
Markel et al.	1993	Y	U	Y	Y	Y	Y	U	Y	Moderate concern	Older reporting standards; history and adverse-event details are incompletely reported.

PRISMA 2020 Checklist

Item	Definition
**Q1**	Demographic characteristics clearly described
**Q2**	Patient history and timeline clearly described
**Q3**	Current clinical condition clearly described
**Q4**	Diagnostic tests or assessment methods and results clearly described
**Q5**	Intervention or treatment procedures clearly described
**Q6**	Post-intervention clinical condition clearly described
**Q7**	Adverse or unanticipated events identified and described
**Q8**	Take-away lesson clearly stated

B. JBI Critical Appraisal Checklist for Case Series (10 items)

Study	Year	Q1	Q2	Q3	Q4	Q5	Q6	Q7	Q8	Q9	Q10	Overall concern	Notes
Xie et al.	2018	Y	Y	Y	U	U	Y	Y	Y	Y	Y	Moderate concern	Eligibility and outcomes are described, but consecutive and complete inclusion are unclear.
Vankipuram et al.	2024	Y	Y	Y	U	U	Y	Y	Y	Y	NA	Moderate concern	Small technical series; consecutive and complete recruitment are unclear.
Moo Young et al.	2024	Y	Y	Y	U	U	Y	Y	Y	Y	Y	Moderate concern	Multicenter retrospective series with variable follow-up and incomplete reporting of recruitment completeness.
Aleissa et al.	2023	Y	Y	Y	U	U	Y	Y	Y	Y	NA	Moderate concern	Clinical data are reported, but consecutive and complete inclusion are not clearly established.
Moo Young et al.	2023	Y	Y	Y	U	U	Y	Y	Y	Y	Y	Moderate concern	Large multicenter series; retrospective selection and heterogeneous neurological follow-up remain concerns.
Luo et al.	2022	Y	Y	Y	U	U	Y	Y	Y	Y	NA	Moderate concern	Small retrospective series with unclear consecutive recruitment and incomplete standardized outcomes.
Obey et al.	2021	Y	Y	Y	U	U	Y	Y	Y	Y	Y	Moderate concern	Objective imaging variables, but retrospective case identification and recruitment completeness are unclear.
Fernández-Fernández et al.	2021	Y	Y	Y	U	U	Y	Y	Y	Y	NA	Moderate concern	Small technical series; completeness of recruitment is unclear.
Tian et al.	2021	Y	Y	Y	U	U	Y	Y	Y	Y	Y	Moderate concern	Defined protocol and outcomes, but non-comparative design and unclear consecutive inclusion.
Erkan et al.	2021	Y	Y	Y	U	U	Y	Y	Y	Y	NA	Moderate concern	Treatment and union outcomes reported; recruitment completeness and standardized neurological follow-up unclear.
Lee et al.	2020	Y	Y	Y	N	N	Y	Y	Y	Y	NA	High concern	Only two patients; non-consecutive and non-complete inclusion cannot be established.
Liu et al.	2021	Y	Y	Y	U	U	Y	Y	Y	Y	Y	Moderate concern	Objective technical outcomes, but retrospective selection and possible learning-curve effects.
Jindal et al.	2022	Y	Y	Y	U	U	Y	Y	Y	Y	Y	Moderate concern	Clinical and radiological outcomes are reported; consecutive complete recruitment remains unclear.
Miyamoto et al.	2020	Y	Y	Y	Y	U	Y	Y	Y	Y	NA	Moderate concern	Explicit eligibility and scheduled follow-up; small retrospective sample.
Chou et al.	2018	Y	Y	Y	U	U	Y	Y	Y	Y	NA	Moderate concern	Small heterogeneous retrospective series with unclear consecutive recruitment.
Tian et al.	2018	Y	Y	Y	U	U	Y	Y	Y	Y	NA	Moderate concern	Detailed operative technique and follow-up; recruitment completeness unclear.
Kanezaki et al.	2019	Y	Y	Y	U	U	Y	Y	Y	Y	NA	Moderate concern	Preliminary small series; complete recruitment and long-term follow-up uncertain.
Park et al.	2012	Y	Y	Y	U	U	Y	Y	U	Y	Y	Moderate concern	Large but heterogeneous series; standardized neurological outcomes and completeness of follow-up are limited.
Ayoub	2012	Y	Y	Y	Y	U	Y	Y	Y	Y	Y	Moderate concern	Eligibility and neurological outcomes described; retrospective design and no comparator.

PRISMA 2020 Checklist

Item	Definition
**Q1**	Clear inclusion criteria
**Q2**	Condition measured in a standard, reliable way
**Q3**	Valid methods used to identify the condition
**Q4**	Consecutive inclusion
**Q5**	Complete inclusion
**Q6**	Clear demographic reporting
**Q7**	Clear clinical information
**Q8**	Outcomes and follow-up clearly reported
**Q9**	Site(s) or clinic(s) clearly reported
**Q10**	Appropriate statistical analysis

PRISMA 2020 Checklist

C. JBI Critical Appraisal Checklist for Cohort Studies (11 items)

Study	Year	Q1	Q2	Q3	Q4	Q5	Q6	Q7	Q8	Q9	Q10	Q11	Overall concern	Notes
Taylor et al.	2025	N	Y	Y	Y	N	Y	Y	Y	U	N	Y	High concern	Groups differed by clinical indication and severity; no adequate adjustment for confounding by indication.
Elsherif and Mokhtar	2023	N	Y	Y	Y	N	Y	Y	Y	U	N	Y	High concern	Direct versus indirect decompression groups were not randomized; baseline neurological severity likely confounded outcomes.
Shi et al.	2023	Y	Y	Y	Y	U	Y	Y	Y	U	U	Y	Moderate concern	Large observational cohort, but retrospective ascertainment and incomplete adjustment for prognostic factors.
Pearson et al.	2018	N	Y	Y	Y	N	Y	Y	Y	U	N	Y	High concern	Open and percutaneous groups were selected clinically; limited adjustment for baseline severity.
Lindahl et al.	2014	Y	Y	Y	Y	Y	Y	Y	Y	Y	Y	Y	Moderate concern	Relatively well-described retrospective cohort with prognostic analysis; residual confounding remains possible.

PRISMA 2020 Checklist

Item	Definition
**Q1**	Groups similar and recruited from the same population
**Q2**	Exposures measured similarly in exposed and unexposed groups
**Q3**	Exposure measured validly and reliably
**Q4**	Confounding factors identified
**Q5**	Strategies to deal with confounding stated
**Q6**	Participants free of outcome at the start
**Q7**	Outcomes measured validly and reliably
**Q8**	Follow-up time sufficient
**Q9**	Follow-up complete and reasons for loss described
**Q10**	Strategies used to address incomplete follow-up
**Q11**	Appropriate statistical analysis
